# ^123^I-MIBG imaging in heart failure: impact of comorbidities on cardiac sympathetic innervation

**DOI:** 10.1007/s00259-022-05941-3

**Published:** 2022-09-08

**Authors:** Paola Gargiulo, Wanda Acampa, Gaetano Asile, Vincenza Abbate, Ermanno Nardi, Federica Marzano, Roberta Assante, Carmela Nappi, Antonio Luca Maria Parlati, Christian Basile, Santo Dellegrottaglie, Stefania Paolillo, Alberto Cuocolo, Pasquale Perrone-Filardi

**Affiliations:** 1 Department of Advanced Biomedical Sciences, University of Naples Federico II, Naples, Italy; 2 IRCCS Synlab SDN, Naples, Italy; 3 Division of Cardiology, Ospedale Accreditato Villa dei Fiori, Acerra, Naples, Italy; 4 Mediterranea Cardiocentro, Naples, Italy

**Keywords:** Heart failure, ^123^I-MIBG, Myocardial sympathetic innervation, Diabetes mellitus, Obesity, Kidney dysfunction, Sleep-disordered breathing

## Abstract

**Purpose:**

Heart failure (HF) is a primary cause of morbidity and mortality worldwide, with significant impact on life quality and extensive healthcare costs. Assessment of myocardial sympathetic innervation function plays a central role in prognosis assessment in HF patients. The aim of this review is to summarize the most recent evidence regarding the clinical applications of iodine-123 metaiodobenzylguanidine (^123^I-MIBG) imaging in patients with HF and related comorbidities.

**Methods:**

A comprehensive literature search was conducted on PubMed and Web of Science databases. Articles describing the impact of ^123^I-MIBG imaging on HF and related comorbidities were considered eligible for the review.

**Results:**

We collected several data reporting that ^123^I-MIBG imaging is a safe and non-invasive tool to evaluate dysfunction of cardiac sympathetic neuronal function and to assess risk stratification in HF patients. HF is frequently associated with comorbidities that may affect cardiac adrenergic innervation. Furthermore, HF is frequently associated with comorbidities and chronic conditions, such as diabetes, obesity, kidney disease and others, that may affect cardiac adrenergic innervation.

**Conclusion:**

Comorbidities and chronic conditions lead to more severe impairment of sympathetic nervous system in patients with HF, with a negative impact on disease progression and outcome. Cardiac imaging with ^123^I-MIBG can be a useful tool to reduce morbidity and prevent adverse events in HF patients.

**Supplementary information:**

The online version contains supplementary material available at 10.1007/s00259-022-05941-3.

## Introduction

**Fig. 1:**
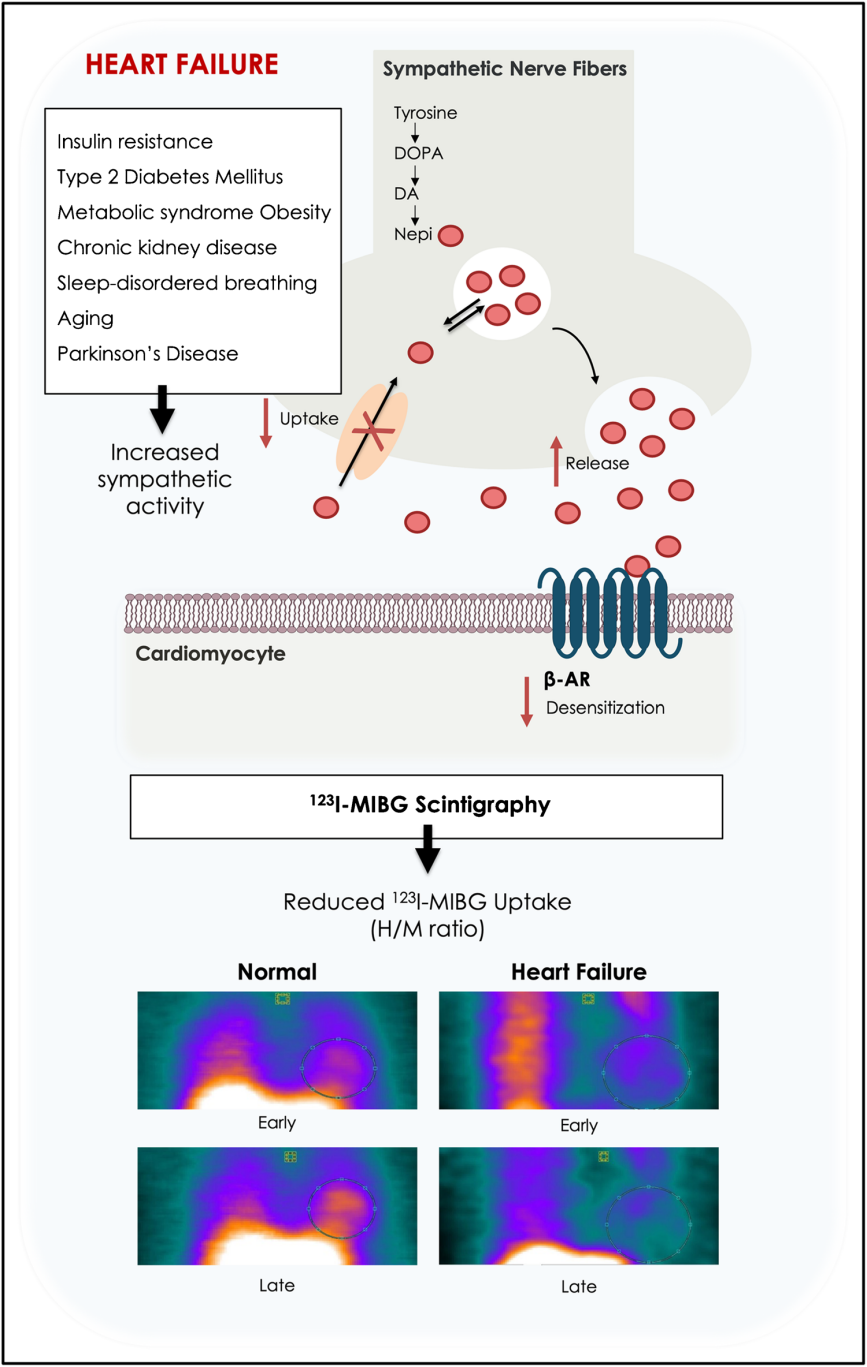
Graphical representation of sympathetic innervation in heart failure. In failing heart, activation of the sympathetic nervous system leads to elevated levels of Nepi, due to increased release and reduced uptake. The result is a persistent activation and subsequent desensitization of β-ARs, which in the long term determines cardiac damage and HF progression. This is usually reflected as decreased cardiac ^123^I-MIBG uptake (reduced H/M ratio) on imaging. Comorbidities and conditions such as aging contribute to impair cardiac adrenergic innervation, with a negative impact on HF progression and outcome. β-ARs, β-adrenergic receptors; DOPA, dihydroxyphenylalanine; DA, dopamine; Nepi, norepinephrine

Heart failure (HF) is a clinical syndrome, characterized by structural and/or functional alterations of the heart resulting in not adequate cardiac output at rest and/or during exercise. To date, the incidence of HF in Europe is about 5/1000 person per year in adults, with a prevalence of 1–2% that increases with age [1]. HF is a primary cause of mortality worldwide, with significant impact on life quality, and extensive healthcare costs. Despite the advances in HF therapies in the past 20 years, long-term prognosis in HF remains poor [2]. In HF patients, the most widely recognized compensatory mechanism is the activation of neurohormonal systems, such as the sympathetic nervous system (SNS) and the renin–angiotensin–aldosterone system (RAAS). Although initially activation of neurohormonal systems improves impaired myocardial function, long-term sympathetic hyperactivity generates additional damage to the heart, thus leading to harmful myocardial remodeling, decline in left ventricular function, and further progression of the disease [3]. Patients with HF display increased levels of norepinephrine (NE), due to altered neuronal release and reuptake [4]. Figure 1 provides a graphical representation of sympathetic innervation in HF.

Imaging with iodine-123 metaiodobenzylguanidine (^123^I-MIBG), a radiolabeled analog of NE, is currently used as a safe and non-invasive tool to evaluate dysfunction of cardiac sympathetic neuronal function and to assess risk stratification and prognosis in HF patients [5]. After injection, ^123^I-MIBG uptake provides, over several hours, a measure of the integrity and function of myocardial sympathetic innervation [2]. Moreover, a strong interplay between cardiac sympathetic dysfunction investigated by ^123^I-MIBG imaging and hemodynamic changes during stress test has been demonstrated in patients with suspected or known coronary artery disease (CAD) undergoing myocardial perfusion imaging [6]. Semiquantitative parameters of ^123^I-MIBG uptake, such as the heart-to-mediastinum (H/M) ratio and washout rate (WR), indicators of autonomic dysfunction, demonstrated prognostic value in patients with HF [7]. As showed by the AdreView Myocardial Imaging for Risk Evaluation in Heart Failure (ADMIRE-HF) study, reduced late H/M ratio and/or increased ^123^I-MIBG washout rate is associated with HF progression, increased incidence of lethal cardiac events, and implantable cardiac device (ICD) discharges [8].

HF is frequently associated with comorbidities and chronic conditions, such as diabetes, obesity, kidney dysfunction, and sleep-disordered breathing (SDB), that may affect cardiac adrenergic innervation, with a negative impact on disease progression and outcome [9]. In addition, cardiac ^123^I-MIBG single photon emission tomography (SPECT) imaging may also provide prognostic data in non-ischemic acute decompensated HF patients and preserved ejection fraction (HFpEF) [10, 11].

The aim of this review is to summarize the most recent evidence regarding the clinical applications of ^123^I-MIBG imaging in patients with HF and related comorbidities.

## Methods

This narrative review involved keyword searches of PubMed and Web of Science. Review search terms included [“MIBG” OR “Sympathetic”] AND [“Heart Failure” OR “HF”] in all fields.

The databases were searched without any restrictions from inception to 01 May 2022. Two authors separately examined the titles and abstracts of all obtained publications to exclude clearly unrelated research. No language restrictions were applied. References of the provided articles were also examined to find out any additional relevant studies. Studies included in the review met the following criteria: (A) published on a peer-reviewed journal; (B) described the impact of ^123^I-MIBG on HF and its comorbidities; and (C) were opinion papers, guidelines, case studies, descriptive studies, randomized control trial, prospective studies, retrospective studies, narrative reviews, and systematic reviews. Search process is shown in Supplementary Fig. 1.

### ^123^I-MIBG imaging in HF associated to insulin resistance and diabetes

Insulin resistance (IR) is a condition characterized by an impaired biologic response of target tissues, such as the liver, muscle, and adipocytes, to insulin stimulation, leading to the compensatory increase in insulin production by beta-cell and impairment of homeostasis systems (hyperinsulinemia, hyperglycemia, hypertension etc.) [12]. Hyperinsulinemia, which is associated to IR, promotes sympathetic neural activity in normal humans [13], and insulin-resistant patients with essential hypertension and normal left ventricular (LV) function have been shown to have impaired cardiac sympathetic innervation [14]. IR progression can lead to the development of type 2 diabetes mellitus (T2DM) that is a well-established risk factor for the incidence of HF [15] and represents a common comorbidity with an estimated prevalence of 30–40% [16]. T2DM is involved in the progression and worsening of symptomatic and asymptomatic HF patients, and it is associated with higher risk of hospitalization and mortality [16]. T2DM is the most common cause of autonomic neuropathy, interesting all types of nerve fibers in the organism. If the parasympathetic system is involved, diabetic patients develop cardiovascular (CV) autonomic neuropathy that is characterized by reduced heart rate variability and/or orthostatic hypotension [17].

The coexistence of HF and IR or diabetes is a poor prognostic factor, and it is associated with a more severe and aggressive HF with reduced ejection fraction (HFrEF), characterized by higher morbidity and mortality [18]. The damaging effects of IR and T2DM in patients with HF are multiple, and they include the direct effects on microvascular circulation, the impairment of regional myocardial blood flow and coronary flow reserve, cellular injuries, dysfunctions in the contraction, and release of muscle cells, and the activation of neurohormonal systems. All these functional, metabolic, and structural alterations contribute to the progression and worsening of HF [19].

Several studies have demonstrated the presence of alteration of cardiac autonomic stimulation in subjects with HF and comorbidities, such as IR and T2DM, with evaluation of the cumulative effect of these comorbidities on cardiac autonomic dysfunction and the possible effect on the stage of the disease and on the prognosis.

Patients with T2DM and without HF have been shown to have a significant reduction in ^123^I-MIBG uptake, most likely based on diabetic neuropathy, which is correlated with worse prognosis [20]. One of the first study reporting the impact of T2DM on cardiac sympathetic activity was conducted in diabetic patients with HF enrolled in the ADMIRE-HF trial. In this population, a reduced ^123^I-MIBG H/M ratio (< 1.6), indicating cardiac sympathetic denervation, was associated with greater HF progression [21]. These evidences emphasize that alterations studied with ^123^I-MIBG imaging are not only descriptive, but they also represent independent prognostic factors. Accordingly, Paolillo et al. reported a significantly increased impairment in cardiac sympathetic innervation in diabetic patients with chronic, severe systolic HF compared to both non-diabetic HF subjects and diabetic patients without HF [22]. An interesting finding from this study was that, among diabetic HF patients, the reduction in cardiac sympathetic activity was correlated to glycemic control, assessed by hemoglobin A1c.

**Fig. 2:**
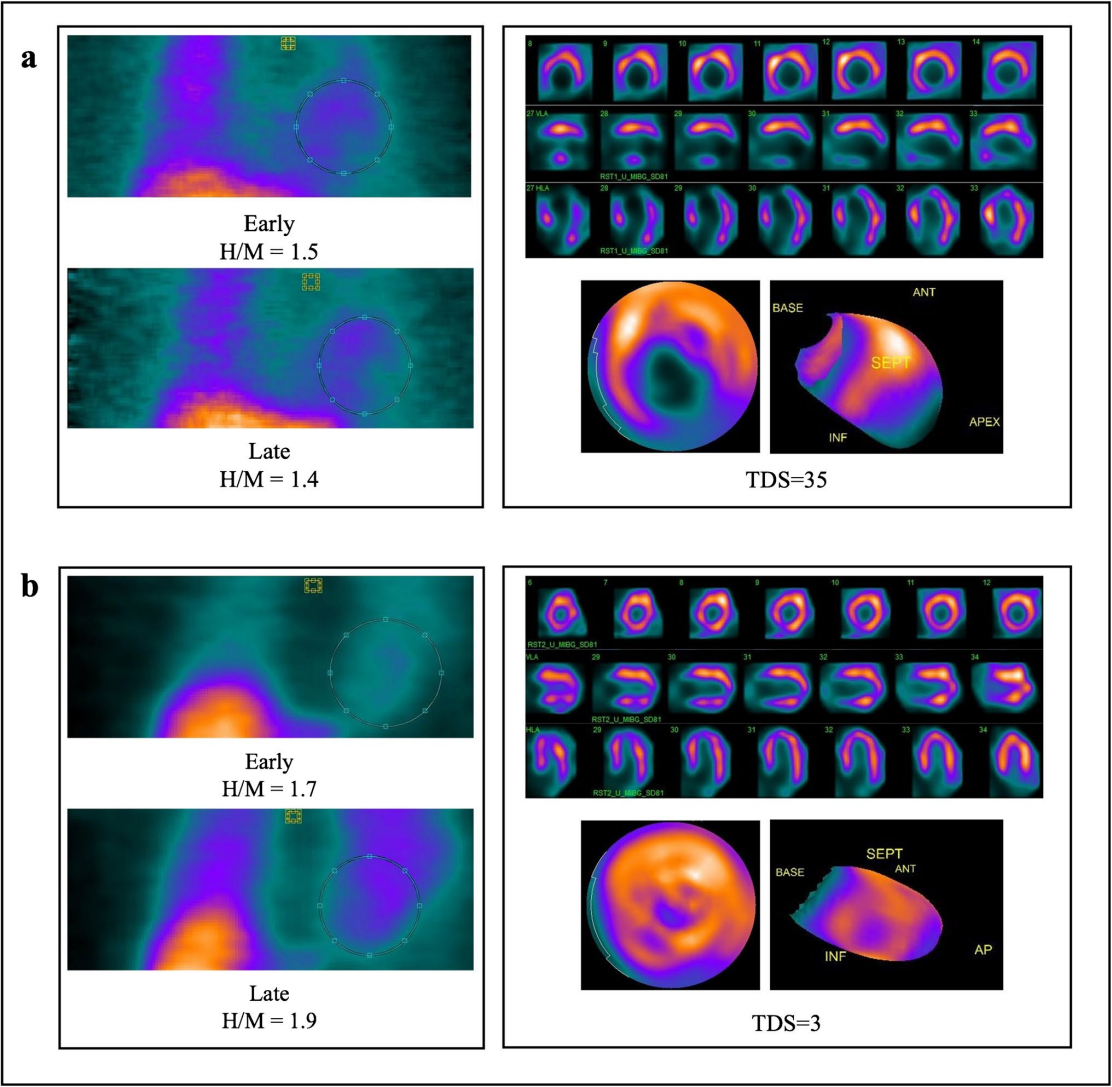
**a** ^123^I-MIBG CZT images of a diabetic patient with HF (LVEF 37%). ^123^I-MIBG H/M ratios obtained from planar equivalent and tomographic images showing an extensive area of reduced innervation in the apex and inferior wall of the left ventricle with total defect score (TDS) of 35. **b** ^123^I-MIBG CZT images of a non-diabetic patient with HF (LVEF 42%). ^123^I-MIBG H/M ratios obtained from planar equivalent and tomographic images showing a mild reduction of innervation in the apex of the left ventricle with total defect score (TDS) of 3

Similar results have been found in a population of non-diabetic HF patients with IR. Compared to matched non-IR subjects, patients with IR showed a significantly reduced early and late H/M ratio, thus suggesting impaired cardiac sympathetic innervation and a more advanced stage of the disease [23]. Although these findings demonstrated a strong interaction between IR, HF, and cardiac SNS, further studies are needed to elucidate the underlying pathophysiological processes. Figure 2a and b show representative examples of ^123^I-MIBG innervation images in a diabetic and non-diabetic patient with HF.

### ^123^I-MIBG imaging in HF associated to metabolic syndrome and obesity

Metabolic syndrome (MS) is a clinical condition with a prevalence of approximately 34% in the general population [24]. It is characterized by a cluster of interrelated metabolic risk factors that are associated with the development of CV diseases, including HF.

In particular, the most widely recognized risk factors that negatively affect disease progression are elevated blood pressure, IR, lipid abnormalities, and obesity [25]. According to the NCEP ATP III criteria, MS occurs if three or more of the following conditions coexist in the same patient: increased waist circumference; elevated triglycerides levels (> 150 mg/dL); reduced high-density lipoprotein (< 40 mg/dL in men or < 50 mg/dL in women); elevated fasting glucose (> 110 mg/dL); blood pressure values over 130/85 mmHg [26].

MS is frequently present in patients with HF, and it is associated with the activation of several molecular, cellular, and neurohormonal pathways that may affect prognosis [19]. IR and obesity represent predominant risk factors associated to the development of MS and are characterized by a marked sympathetic overactivity related to increase HF through several different mechanisms [27].

Several evidences have clearly shown that sympathetic activity is increased in human obesity.

**Fig. 3:**
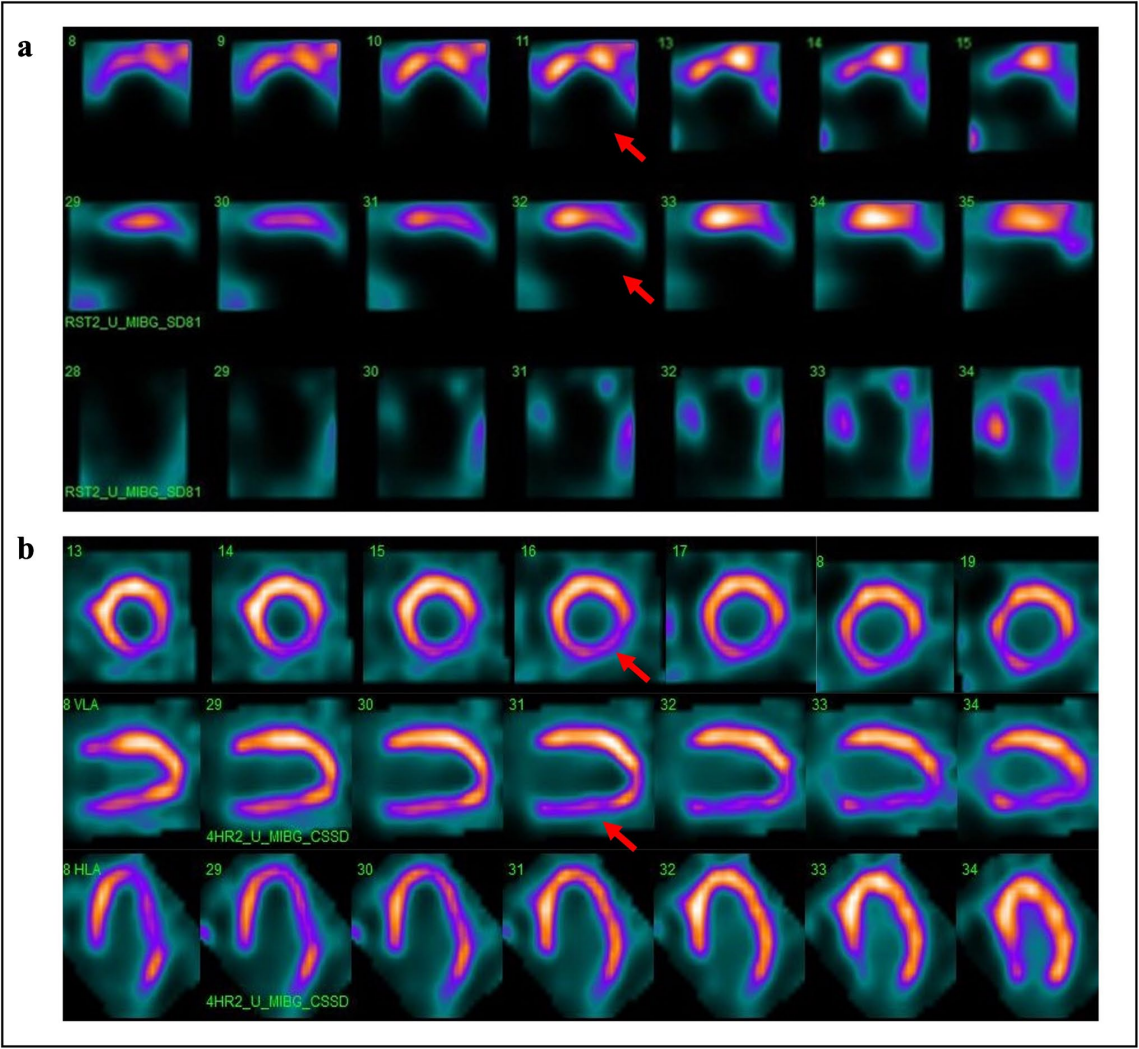
**a** ^123^I-MIBG CZT images of a patient with HF (LVEF 32%) with obesity. An extensive area of reduced innervation in the apex, antero-septal, and inferior wall of the left ventricle was visible (red arrows). **b**.^123^I-MIBG CZT images of a patient with HF (LVEF 35%) without obesity. A mild reduction of innervation in the infero-lateral wall of the left ventricle was visible (red arrows)

A study conducted by Pellegrino and coworkers investigated the impact of obesity and the influence of the acquisition protocol on ^123^I-MIBG imaging indexes of cardiac sympathetic innervation, in patients with HF. They found a significant reduction of early and late H/M ratios in obese HF patients, compared to non-obese subjects, both in supine and prone positions [28]. A significant reduction in cardiac adrenergic innervation has been also reported by Komici et al. in obese patients with HF. Results of this study revealed that BMI, together with age and LVEF, was significantly correlated with reduced early and late H/M ratios in HF [29]. Recent studies have also indicated a close interdependence between epicardial adipose tissue (EAT), which represents the visceral fat depot of the heart, and myocardial autonomic function. In this context, a recent study by Parisi et al. explored the relationship between EAT thickness and sympathetic activity, assessed by cardiac ^123^I-MIBG imaging. In particular, the authors demonstrated that, in patients with systolic HF, increased EAT thickness represents a source catecholamines production and is correlated to cardiac sympathetic denervation and disease progression [30]. Furthermore, abdominal obesity but not general obesity has been associated with low early and late H/M ratio in HF patients with preserved ejection fraction. These results suggest that abdominal obesity is involved in sympathetic nerve abnormalities related to HF and loss of visceral fat should be considered part of a multifactorial and multidisciplinary strategy in the treatment of HF patients [31]. Figure 3a and b show representative examples of ^123^I-MIBG images in HF patients with (BMI = 44.1) and without obesity **(**BMI = 22).

### ^123^I-MIBG imaging in HF associated to chronic kidney disease

Cardiac and renal diseases are common conditions that frequently coexist in the same patient, thus significantly increasing morbidity, mortality, and the healthcare cost [32]. Chronic kidney diseases are widely recognized risk factors for the incidence and progression of CV diseases and HF [33]. Impairment in kidney function, defined by reduced creatinine clearance and glomerular filtration rate (GFR), has been associated with heightened risk of hospitalization and death in patients with chronic HF with both preserved and reduced ejection fraction [34]. The term cardio-renal syndrome is usually referred to a pathophysiological condition characterized by combined cardiac and renal dysfunction. The excessive activation of neurohormonal compensatory mechanisms, such as the SNS and the RAAS, has been implicated in many ways in the progression of cardio-renal syndrome, thus contributing to adverse outcomes in these patients [35]. As previously mentioned, HF is characterized by reduced cardiac autonomic innervation, which can be visualized by ^123^I-MIBG myocardial scintigraphy. Notably, in patients with renal dysfunction, sympathetic hyperactivity has been also reported and it has associated with tubular damage, reactive oxygen species formation, and renal fibrosis [32].

In this context, several studies have been conducted to evaluate the relationship between renal diseases and cardiac adrenergic impairment, assessed by ^123^I-MIBG imaging. In chronic HF patients, impaired cardiac sympathetic innervation assessed by ^123^I-MIBG activity has been associated with reduced kidney function and increased risk of cardiac death [36]. Accordingly, Verschure and colleagues investigated the different predictive potentials of ^123^I-MIBG imaging and GFR on outcomes in patients with chronic HF and found that ^123^I-MIBG scintigraphy was a better predictor of cardiac death, compared to the evaluation of renal function [37]. In a subsequent study, Malhotra et al. found a prevalence of cardiac sympathetic dysfunction in patients with both HF and renal dysfunction. However, among HF patients in the same NYHA class, no difference was observed in terms of H/M ratio between patients with and without renal dysfunction. Of note, patients with highly impaired renal function were not included in that study [38]. More recently, Marsico et al. showed that, in a population of 263 patient with mild-to-severe HF, ^123^I-MIBG uptake was significantly reduced in HF patients with severely impaired renal function compared to HF subjects with preserved renal function [39]. Furthermore, the combination of chronic kidney dysfunction and impaired cardiac sympathetic nervous activity, assessed by ^123^I-MIBG scintigraphy, has been shown to have a high prognostic value in the prediction of lethal arrhythmic events in HF patients [40]. In conclusion, these results suggest that impaired cardiac sympathetic innervation might contribute to worsen prognosis in patients with HF and concomitant renal dysfunction.

### ^123^I-MIBG imaging in HF and aging

Cardiac dysfunction and HF are very common in the elderly population, thus drastically affecting survival rate and quality of life. A salient characteristic of the failing heart, as well as the aged heart, is the deregulation of the SNS that is associated to disease progression and poor prognosis [41].

^123^I-MIBG imaging has been widely used to assess cardiac sympathetic innervation and to predict the prognosis, in both HF and aging. Physiological aging is characterized by impaired CV function and SNS hyperactivity, which results in increased circulating levels of catecholamines and decreased cardiac β-adrenergic receptor responsiveness in the elderly population [42]. Several studies have investigated the effect of age on cardiac innervation by assessing myocardial ^123^I-MIBG uptake, and they have reported conflicting results. It has been demonstrated that ^123^I-MIBG uptake is inversely related to age in subjects without CV disease [43, 44], suggesting that age should be considered when assessing cardiac innervation with ^123^I-MIBG imaging. Conversely, a more recent study by Jacobson and colleagues has failed to find significant correlation between age and cardiac ^123^I-MIBG uptake in old healthy individuals [45]. In 2016, Rengo et al. investigated for the first time the impact of age on cardiac sympathetic innervation in patients with systolic HF. In a population of 180 HF patients, the authors found a significant reduction in ^123^I-MIBG uptake in elderly compared to younger patients [46]. These findings suggest that the age-related effects on cardiac sympathetic innervation should be considered in patients with HF.

### ^123^I-MIBG imaging in HF associated to SDB

Previous studies have demonstrated that SDB is a common comorbidity in patients with HF, both in form of obstructive sleep apnea (OSA) and central sleep apnea (CSA), with a significant impact on disease progression and prognosis. Adverse effects of SDB in HF are mostly mediated by increased sympathetic stimulation that contributes to high morbidity and mortality rates [47]. In patients with OSA, disordered breathing events, including recurrent apneas, hypoxia, and arousal, are associated to increased chemoreflex-mediated adrenergic outflow that persists also into the daytime [48]. Similarly, in patients with HF and CSA, a further increase in resting sympathetic activity has been reported during apnea episodes that is associated to reflex hyperventilation [49].

In addition, it has been demonstrated that ventilatory therapy is able to reduce SNS activity and improve prognosis in patients with HF and SDB [50]. Scala et al. demonstrated that patients with chronic systolic HF showed high prevalence (77%) of SDB, and this condition was associated with significant lower values of H/M ratios, thus suggesting a chronic increase in cardiac sympathetic stimulation [51]. Furthermore, in this study SDB and cardiac sympathetic innervation parameters contributed to predict CV outcome and HF hospitalization; indeed, a worse prognosis was observed in patients with altered H/M ratio and moderate–severe SDB [51]. Accordingly, adaptive servo-ventilation, a novel therapy for sleep disorders, has been shown to improve cardiac function in HF patients. Indeed, in ^123^I-MIBG imaging early H/M ratio was increased after 6 months of treatment with adaptive servo-ventilation, without changing the washout rate [52]. Thus, the present findings underline an important contribution of SDB to adrenergic impairment in HF. Therefore, assessment of SDB should be included in the evaluation of patients with HF, in order to better predict clinical outcome.

### ^123^I-MIBG imaging in HF and Parkinson’s disease

Parkinson’s disease (PD) is the second most common neurodegenerative disease, affecting approximately 1% of the elderly population, with a prevalence increasing with age [53].

At a molecular level, PD is characterized by the degeneration of dopaminergic nerve cells in the substantia nigra and by the accumulation of α-synuclein in Lewy bodies [53].

HF has been found to be a common comorbidity and the third leading cause of death in PD patients. Nevertheless, the causes of increased prevalence of HF in patients suffering from PD are currently unknown [54]. In this context, several studies have been conducted to evaluate the prognostic and diagnostic role of cardiac ^123^I-MIBG imaging in the diagnosis of PD, especially in the early stages [55]. Indeed, cardiac ^123^I-MIBG uptake has been shown to be reduced in patients with Lewy body diseases, including PD, thus representing a useful tool to differentiate PD from other parkinsonisms [56].

The standardized technique for ^123^I-MIBG scintigraphy includes the evaluation of an early and delayed acquisition (15 min and 4 h after injection). Recently, Frantellizzi et al. demonstrated a potential role of an immediate (5 min) or early (15 min) planar ^123^I-MIBG imaging in patients with PD. This result was not found in patients with HF, in which late acquisition was confirmed to be the best timing for image interpretation, thus underlying the different pathophysiology of these diseases [57].

Clinical manifestations of PD comprise a wide spectrum of non-motor symptoms, including CV autonomic dysfunctions [58]. Cardiac sympathetic denervation, assessed by ^123^I-MIBG imaging, has been found in patients with PD since the early stages of the disease and might have a negative impact on long-term CV outcome [59, 60]. These results suggest that assessment of myocardial sympathetic innervation through ^123^I-MIBG imaging can have a prodromal role in the identification of early PD.

### Multiple comorbidities and cardiac innervation

Aging and several comorbidities, including T2DM, obesity, CKD, and SDB, are known to be independently associated with reduced cardiac sympathetic innervation. However, a meta-analysis by Steinberg and colleagues has analyzed the efficacy of ICD therapy in patients with systolic HF and related comorbidities, demonstrating that, in the presence of significant comorbid illness, the benefit of this therapeutic approach on survival is considerably attenuated [61]. In accordance, it has been shown that in patients with systolic HF, benefit of primary therapy with ICD, in terms of survival, was limited in the high-risk groups [62]. A recent study of Bencivenga et al. documented that the number of concomitant pathologies does not influence ^123^I-MIBG parameters of cardiac innervation, including late H/M ratio, as it is evident from the results of the regression analysis, which also included age, gender, BMI, and LVEF as independent variables [63]. However, a further study by Kayama et al. has shown that cardiac ^123^I-MIBG imaging could provide additional prognostic information over the comorbid burden, in patients with acute decompensated HF [64].

**Fig. 4:**
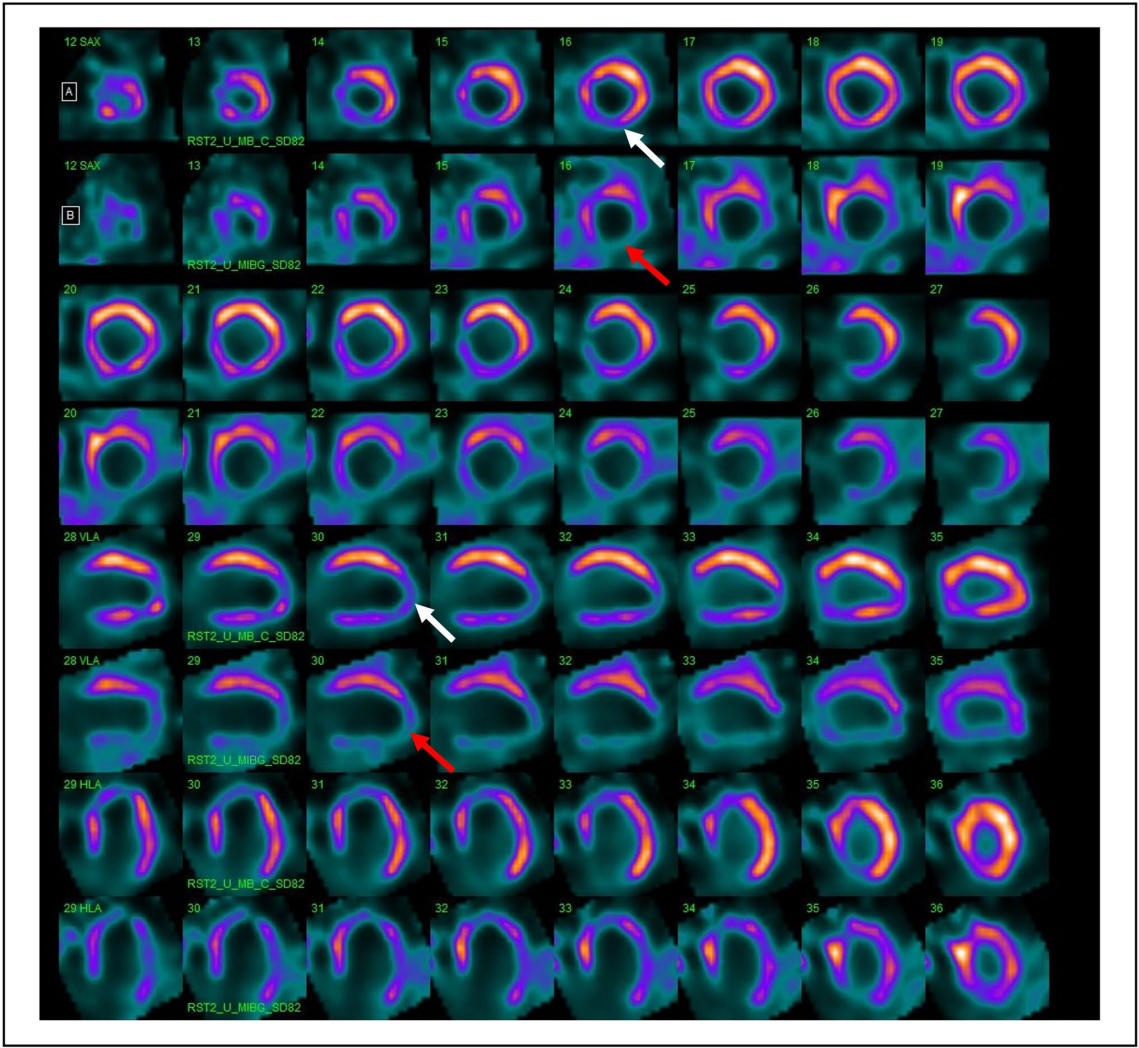
^99m^Tc-sestamibi (**A**) and ^123^I-MIBG (**B**) CZT images of a patient with HF (LVEF 47%). An area of partially preserved perfusion in the ^99m^Tc-sestamibi images (white arrows) but reduced innervation in the ^123^I-MIBG images (red arrows), in the apex and infero-lateral wall of the left ventricle (mismatched area 10%) was visible

Since cardiac ^123^I-MIBG uptake has been demonstrated to improve the cost-effectiveness screening of ICD guideline–eligible HF patients and risk stratification of high-risk patients, aging and comorbidities are variables that may potentially identify those HF patients at highest risk. It should be considered that low-dose assessment of simultaneous perfusion and innervation imaging [65] could provide the possibility to evaluate the mismatch perfusion/innervation area as an important variable in the identification of high-risk patients. A representative example of ^99m^Tc-sestamibi/^123^I-MIBG dual isotope acquisition in a patient with HF and mild reduction of LVEF is shown in Fig. 4. In this patient, a mismatch area involving 10% of the left ventricle was observed, as index of impaired sympathetic innervation and preserved perfusion. The measurement of perfusion/innervation mismatch represents a key step in the prognosis assessment, as it has been demonstrated that these mismatch areas are triggers of ventricular arrhythmias and may be used as potential therapeutic targets. However, several evidences indicate that HF patients with multiple comorbidities are at high risk of all-cause mortality but are less likely to die for sudden cardiac death (SCD) and/or to receive benefits form ICD therapy. Therefore, since myocardial denervation is known to increase the arrhythmic risk in HF patients, these results may be consistent with the observation that an elevated number of comorbidities attenuates the benefit of ICD therapy in HF patients.

## Discussion

^123^I-MIBG imaging has been proven to be a useful and non-invasive tool to evaluate cardiac innervation impairment, to assess disease progression, and to stratify prognosis in patients with HF and related comorbidities. In recent years, research has been focused on the use of this tool to diagnose, evaluate the stage, and understand the pathophysiological processes underlying HF. The current guidelines recommend ICD implantation for the prevention of SCD in patients with HF and significantly reduced LVEF (< 35%) [1]. However, implantation of these devices does not improve cardiac function or symptoms and creates an additional burden for recipient patients, including a series of device-related complications and adverse events. On the other hand, current indication to ICD implantation does not adequately identify some patients with a moderate reduction of LVEF (> 35%) at risk for SCD [66]. In this context, cardiac ^123^I-MIBG imaging has emerged as a helpful tool in the screening of HF patients eligible for ICD implantation and in the prediction of subjects who might not benefit from device implantation [67]. Recently, Nakajima and coworkers proposed a model based on patients with documented 2-year outcomes of HF incorporating ^123^I-MIBG imaging results to differentially predict risk of life-threatening arrhythmic events and HF death providing a more successful selection of therapeutic strategies tailored to individual patient risk status [68]. A recent systematic review by Pontico et al. evaluated the prognostic value of ^123^I-MIBG scintigraphy in assessing risk stratification concerning arrhythmic event and SCD in patients with HF. Data from this study suggest that patients with increased washout rate and reduced ^123^I-MIBG myocardial uptake have a worse prognosis, with increased risk of developing arrhythmic event and SCD [5]. These evidences indicate that ^123^I-MIBG could be a useful tool to evaluate the prognosis and stratify the risk of cardiac events in HF patients, especially in those with multiple comorbidities that could worsen cardiac sympathetic innervation. Although cardiac ^123^I-MIBG is the most studied adrenergic radiotracer, other positron-emitting radiotracers have been investigated to characterize cardiac SNS and to improve image quality in positron emission tomography (PET) [69, 70]. Therefore, cardiac imaging with ^123^I-MIBG and analogous PET tracers can play an important role in reducing morbidity and preventing adverse events in HF patients. However, additional studies are needed to better assess the role of SNS imaging in clinical management of patients with HF.

## Conclusions

In clinical practice, HF is frequently associated with comorbidities such as diabetes, MS, obesity, kidney dysfunction, and SDB that unfavorably impact on prognosis.

As summarized in this review, the coexistence of these chronic conditions is associated with more severe impairment of SNS that may, at least in part, mechanistically explain this association. These observations pave the way to future studies aiming at assessing the clinical value of neuroadrenergic imaging in patients with HF.

## Supplementary information

Below is the link to the electronic supplementary material.

Supplementary file1 (DOCX 45 KB)
