# Supplementary material for: 123I-MIBG imaging in heart failure: impact of comorbidities on cardiac sympathetic innervation
Source: Eur J Nucl Med Mol Imaging. 2022 Sep 8;50(3):813–24. doi: 10.1007/s00259-022-05941-3 (PMC9852124; doi:10.1007/s00259-022-05941-3)
Supplement: Supplementary file 1 — Supplementary file1 (DOCX 45 KB) [file 259_2022_5941_MOESM1_ESM.docx]

**Identification of studies via databases and registers**

Records removed *before screening*:

Duplicate records removed (n = 11936)

Retractions (n = 21)

Records identified from:

Pubmed (n = 9775)

Web of Science (n = 13353)

**Identification**

Records excluded after title/abstract screening (n = 11069)

Records screened

(n = 11171)

Reports sought for retrieval

(n =102)

Reports not retrieved

(n = 0)

**Screening**

Reports assessed for eligibility

(n =102)

Reports excluded for not fulfilling inclusion criteria (n = 31)

Studies included in review

(n = 71)

**Included**

**Supplementary Fig. 1** Flow diagram for the selection of the included studies.
